# Examining the sustainability and effectiveness of co-created physical activity interventions in vocational education and training: a multimethod evaluation

**DOI:** 10.1186/s12889-022-13133-9

**Published:** 2022-04-15

**Authors:** Eva Grüne, Johanna Popp, Johannes Carl, Jana Semrau, Klaus Pfeifer

**Affiliations:** Department of Sport Science and Sport, Friedrich-Alexander University Erlangen-Nürnberg, Gebbertstraße 123b, 91058 Erlangen, Germany

**Keywords:** Participation, Participatory approach, Implementation, Maintenance, Health promotion, Adolescents, Apprentices, Pragmatic evaluation approach

## Abstract

**Background:**

Co-creation approaches are increasingly used in physical activity promotion to develop interventions tailored to the target group and setting. The resulting complexity of such interventions raises challenges in evaluation. Accordingly, little is known about the effectiveness of co-created interventions and the underlying processes that impact their sustainable implementation. In this study, we attempt to fill this gap by evaluating co-created multi-component physical activity interventions in vocational education and training in nursing care and automotive mechatronics regarding (1) their sustainable implementation at the institutional level and (2) the effectiveness of single intervention components at the individual level.

**Methods:**

Following a multimethod design, we conducted a questionnaire survey (*n* = 7) and semi-structured interviews (*n* = 4) to evaluate the sustainability of the interventions. Quantitative data were analyzed descriptively, and qualitative data were analyzed using qualitative content analysis. To examine the interventions’ effectiveness, we conducted two non-randomized controlled trials (*n* = 111). Analysis of variance was used to examine differences between groups.

**Results:**

At the institutional level, long-term implementation of single intervention components in nursing care was observed; in contrast, long-term implementation in automotive mechatronics was not observed. In this context, various factors at the outer contextual (e.g., COVID-19 pandemic), inner contextual (e.g., health-promoting leadership), intervention (e.g., acceptance), and personal levels (e.g., champion) influenced sustainability. At the individual level, no significant intervention effects were found for changes in physical activity behavior and physical activity-related health competence.

**Conclusion:**

The role of co-creation on the effectiveness and sustainability of physical activity promotion in vocational education and training cannot be answered conclusively. Only in the nursing care sector, a co-creation approach appeared promising for long-term intervention implementation. Sustainable implementation depends on various influencing factors that should be considered from the outset. Demonstrating effectiveness at the individual level was challenging. To conclusively clarify both the role and impact of co-creation, methodologically complex and elaborate evaluation designs will be required in future research projects.

**Trial registration:**

This study was retrospectively registered at clinicaltrials.gov on 24/08/2021 (NCT05018559).

**Supplementary Information:**

The online version contains supplementary material available at 10.1186/s12889-022-13133-9.

## Background

For many young people, vocational education and training (VET) represents the first step toward working life. Despite their recent entry into the labor market, young workers are already exposed to increased health risks and are vulnerable to work-related diseases [1]. The health burdens of apprentices appear high in physically demanding occupations, such as in the automotive industry or the nursing care sector [2, 3]. Concurrently, insufficient physical activity (PA) behavior among apprentices has been reported [4]; this also applies to the automotive mechatronics and nursing care sectors [2, 5]. Although there is incontrovertible evidence of the lifelong health benefits of PA underlining the need for PA-promoting interventions [6, 7], these interventions are lacking in the field of VET [8].

In contrast to the overwhelming evidence on the benefits of PA, there is only limited evidence on the effectiveness of different intervention strategies for promoting PA [9, 10]. When developing interventions to promote PA among individuals, it is essential to understand why some people are more physically active than others [11]. The ecological model by Bauman et al. [12] provides a comprehensive framework to explain PA, suggesting that determinants at the individual, behavioral, social, environmental, and political levels play a contributing role in PA. Accordingly, the successful promotion of PA demands the consideration of different influencing factors at different levels [12]. Hence, the interaction between the individual and environmental levels also comes forward, as an effective behavioral change requires supportive environments and policies [13].

However, the success of an intervention relies on both effectiveness and sustained implementation. To achieve lasting intervention effects at the individual level, long-term implementation of the intervention at the institutional level is fundamental [14]. The extent to which interventions are maintained depends on different factors relating to the innovation itself (e.g., fit, adaptability, effectiveness), the context (e.g., climate, culture, leadership), the capacity (e.g., champions, funding, resources), and processes and interactions (e.g., engagement, shared decision-making, partnership) [15].

To ensure both the effectiveness and sustainability of interventions, co-creation approaches in which researchers develop interventions alongside relevant stakeholders seem to be promising. Co-created interventions can be tailored to the target group and given setting, allowing for the development of localized solutions [16, 17]. By involving the target group and listening to their voices, relevant determinants of PA can be identified and addressed [18–20], thereby increasing the acceptability and effectiveness of an intervention [21, 22]. Moreover, a co-creation approach can facilitate contextualization of the new intervention for the specific setting by embedding it into established routines and structures, utilizing existing resources, and building new capacities [23, 24, 18]. The specific adaptations of the intervention to the setting promote the routinization of the intervention, which in turn increases the likelihood of its sustained implementation [25–28].

Against this background, the research project Physical Activity-related Health Competence in Apprenticeship and Vocational Education (PArC-AVE), embedded in the research consortium of Capital4Health, addressed PA promotion in VET in the automotive mechatronics and nursing care sectors. The primary aim of the project was to develop and implement PA-promoting interventions tailored to the needs of the target group and the given setting in two German VET institutions using a co-creation approach involving members of the target group and other relevant actors from research, policy, and practice. During the participatory development and implementation of interventions, the focus was on both the individual level by promoting apprentices’ PA and physical activity-related health competence (PAHCO) [29], and on the institutional level by building capacities for a PA-friendly environment. A first evaluation of the project showed that the co-creation approach succeeded in developing and implementing PA-promoting interventions and thus in building new capacities for PA promotion on the institutional level [30]. However, the effectiveness and sustained implementation of these interventions have not been studied yet.

As interventions become more complex (in this case, co-created multi-component interventions tailored to the target group and the given context), their evaluation also becomes more challenging [31]. When evaluating complex interventions, it is important to know whether they work and how and why they work [32]. Therefore, evaluating both the effectiveness of the intervention and the context, including the underlying processes and factors affecting implementation, is recommended [31, 16, 33]. To cope with this complexity, pragmatic evaluation approaches characterized by theoretical flexibility, methodological comprehensiveness, and operational practicality are increasingly used [34–36]. In this context, both qualitative and quantitative research methods are applied, as they answer different research questions on the one hand and provide a comprehensive evidence base by combining methods and data triangulation on the other hand [34, 37, 38].

Despite the growing popularity of co-creation approaches in developing interventions, the long-term evaluation of both the outcomes and underlying processes of these interventions is sparse [22]. Within the research project PArC-AVE, we aimed to address this issue and evaluated (1) the sustainable implementation of the multi-component interventions developed at the institutional level and (2) the effectiveness of specific components of these complex interventions at the individual level.

## Methods

### Overall study design

This study was based on previous research using a co-creation approach called cooperative planning [39, 40] to develop and implement PA-promoting interventions. Therefore, two separate cooperative planning processes were conducted from July to December 2016 and resulted in the development of two multi-component interventions comprising various intervention components to promote apprentices’ PA and PAHCO (see Additional file 1; [30]); the subsequent implementation of the interventions was the project partners’ responsibility. A detailed overview of the cooperative planning processes and the developed interventions can be found elsewhere [30].

**Fig. 1:**
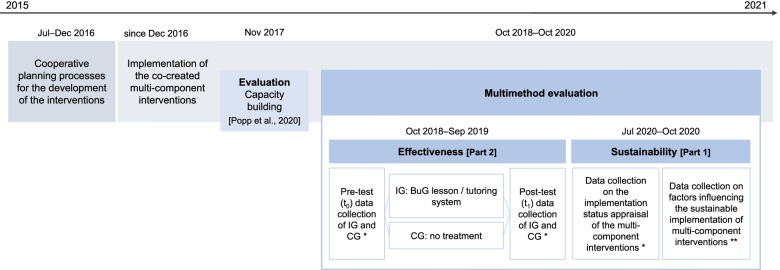
Overall study procedure of the PArC-AVE project, including the multimethod evaluation of sustainability and effectiveness. BuG = Ger. “Bewegt und Gesund”, Eng. physical activity and health); CG = control group; IG = intervention group; * quantitative data collection using standardized questionnaires; ** qualitative data collection using semi-structured interviews

In this study, we used a multimethod design triangulating qualitative and quantitative data [41, 42] to facilitate a more comprehensive evaluation of the PArC-AVE project, focusing on interventions’ sustainability and effectiveness. To evaluate the sustainability of the multi-component interventions (Part 1), we conducted a short questionnaire survey in July 2020 on the current implementation status and appraisal of the single intervention components. Moreover, we conducted semi-structured interviews from September to October 2020 to identify factors influencing the long-term implementation of the multi-component interventions. To examine the effectiveness of single components of the multi-component interventions (Part 2), we conducted two non-randomized controlled trials from October 2018 to September 2019. Figure 1 shows the overall study procedure of the PArC-AVE project, including the evaluation of the sustainability and effectiveness of interventions using a multimethod design. To ensure comprehensive reporting, we used the Standards for Reporting Implementation Studies (StaRI) checklist [43] (see Additional file 2) and the Consolidated Criteria for Reporting Qualitative Research (COREQ) checklist [44] (see Additional file 3).

### Part 1: Sustainability

#### Setting and participants

The first part of this study was undertaken in two German VET institutions where the cooperative planning processes for the development and implementation of PA-promoting interventions had previously been conducted [30]. The two VET institutions differed because the setting in the nursing care sector was a school, and in the automotive mechatronics sector, it was a company. Referring to the first evaluation of the cooperative planning processes [30], all interviewees of the past evaluation and additionally the current project partners’ contact persons were initially invited to participate in a short questionnaire survey on the current implementation status of the interventions developed. Following the purposeful sampling of information-rich cases [45], the interviewees were subsequently selected according to the results of the first evaluation and the previous questionnaire survey, and were invited to participate via email or telephone. In total, seven participants gave their written consent and participated in the questionnaire survey, and four participants, two each from the automotive mechatronics and nursing care institutions, participated in the interviews (see Table 1).

**Table 1:** Setting and participants of the sustainability evaluation

	**Automotive mechatronics**	**Nursing care**
**Implementation status and appraisal of interventions** [questionnaire survey]		
Participants invited	^a^Coordinator for apprenticeship projects [f]; Director of the VET center [m]; Head of the automotive education sector [m]; Instructor [m]; ^a^Occupational physician [f]; ^a^Member of the works council [m]; Youth apprentices’ representative [m]	^a^Headmaster [m]; ^a^Head of the nursing education program [m]; ^a^Head of the school subject of nursing [m]; ^a^Teacher [f]; Teacher [m]; Member of the works council of the hospital [f]
**Factors influencing sustainability** [interviews]		
Participants invited	^a^Coordinator for apprenticeship projects [f]; ^a^Member of the works council [m]	^a^Head of the nursing education program [m]; ^a^Head of the school subject nursing [m]

*f* female, *m* male, *VET* vocational education and training

^a^participants agreed to participate

#### Data collection

The data collection was performed in two steps. First, the current implementation status and an initial appraisal of the multi-component interventions’ single components were assessed by a self-developed questionnaire with eight items per intervention component in July 2020. An exemplary item on the appraisal of intervention components was, “The intervention component is tailored to the needs and requirements of the apprentices,” with higher scores on a five-point Likert scale indicating a higher agreement.

Second, semi-structured interviews were conducted to evaluate the factors influencing the sustainable implementation of interventions from September to October 2020. We developed a theory-based interview guide based on the existing literature. The interview guide comprised open-ended questions addressing the following factors of sustainability: innovation, context, capacity, and processes and interactions [15, 46]. An exemplary question on the factors influencing sustainability was, “What has contributed to the fact that some of the intervention components are still taking place, but others no longer?” One author (EG; research associate, Master of Arts degree, female) conducted the interviews exclusively with the interviewees either at their workplace or online using the teleconferencing software Zoom Cloud Meetings (Zoom Video Communications, Inc., San Jose, USA). Besides collaboration within the research project, there was no relationship between the interviewer and the interviewees. The interviews were audio-recorded and lasted, on average, 32.25 min (*SD* = 13.30; range 20–51 min).

#### Data analysis

Questionnaire data were analyzed using Microsoft Excel (Microsoft, Redmond, USA). Descriptive statistics were generated to illustrate the current implementation status and to overview the appraisal of intervention components. The interviews were transcribed verbatim. For anonymity reasons, we replaced personal names with working positions and company, institution, and city names with pseudonyms. The transcripts were analyzed using qualitative content analysis [47]. The structuring content analysis comprised the following steps: (i) initiating text work, (ii) forming main categories deductively based on the interview guide, (iii) coding data with the main categories, (iv) compiling all coded text passages of the main categories, (v) forming subcategories inductively based on the transcribed material, (vi) coding all data using the refined coding frame, and (vii) evaluating and interpreting [47]. Two authors (EG and JP) independently coded all interview transcripts. For discrepancies between coders, consensus was reached through discussions. The software MAXQDA Plus 2020 (VERBI Software, Berlin, Germany) was used for data coding and analysis.

### Part 2: Effectiveness

#### Setting and participants

The second part of this study was conducted at four VET institutions in Southern Germany – two each in the automotive mechatronics and nursing care sectors. Following a pragmatic evaluation approach, the two intervention groups were recruited from individuals within the two automotive mechatronics (IG-A) and nursing care (IG-N) institutions that conducted the cooperative planning processes to develop the interventions and also participated in the evaluation of sustainability (see part 1). The two control groups were established by recruiting apprentices in comparable automotive mechatronics (CG-A) and nursing care (CG-N) institutions that did not previously participate in the co-creation approach. Informed written consent was obtained from all participating apprentices. In total, 111 first-year apprentices from the automotive mechatronics and nursing care sectors, with an average age of 18.39 years (*SD* = 3.12), participated in this study. Table 2 shows further information on the final sample.

**Table 2:** Baseline characteristics of participants included in the effectiveness analysis

	**Automotive mechatronics**		**Nursing care**	
	**IG-A**	**CG-A**	**IG-N**	**CG-N**
Sample size, *n*	23	37	17	34
Gender (male), *n* (%)	14 (60.9)	30 (81.1)	5 (29.4)	3 (8.8)
Age (years), mean (*SD*)	17.30 (1.11)	18.00 (1.60)	20.47 (6.58)	18.50 (2.16)
Body mass index, mean (*SD*)	22.43 (3.42)	23.65 (4.22)	26.52 (5.74)	21.85 (2.62)

*IG-N* intervention group nursing care, *CG-N* control group nursing care, *IG-A* intervention group automotive mechatronics, *CG-A* control group automotive mechatronics

#### Intervention

In this study, we focused on the evaluation of the tutoring system in the automotive mechatronics institution and the BuG lesson (German: “Bewegt und Gesund”; English: PA and health) in the nursing care institution, both as central parts of the multi-component interventions (see [30]).

In the tutoring system, automotive mechatronics apprentices act as PA promotors for their colleagues. In a peer-to-peer approach, apprentices voluntarily participated in a workshop to be qualified as tutors for PA promotion. Subsequently, the tutoring system occurs every two weeks for about 10 to 15 min during regular working hours, allowing the tutors to discuss the issues of PA and health among their peers. The BuG lesson comprises a weekly 90-min lesson for nursing care apprentices during regular school hours, covering PA and health in theory and practice. Previously, the nursing teachers voluntarily underwent a qualification process as PA instructors, enabling them to conduct BuG lessons independently. Both intervention components had in common that the individuals delivering these intervention components (i.e., tutors and teachers) had previously undergone a qualification process. The contents covered in the qualification processes were based on behavior change techniques (e.g., behavioral practice, goal-setting, instructions on how to perform a behavior [48]), which were modified specifically for the target group of apprentices and recorded in a manual that was provided to the instructors to deliver the intervention.

Thus, the apprentices of IG-A received the tutoring system, and the apprentices of IG-N participated in the BuG lesson, while apprentices of both control groups did not participate in any treatment in addition to their VET curriculum.

#### Data collection

Data were collected prior to the intervention at the beginning of the VET year (t_0_: October 2018–March 2019) and at the end of the VET year (t_1_: June–September 2019) using a standardized questionnaire. To assess apprentices’ PA levels, the validated Physical Activity, Exercise and Sport Questionnaire was used [49]. This instrument distinguishes between leisure/transport activities (eight dimensions) and sport/exercise activities (up to three free specifications) by reporting the frequency and duration of the participants’ activities in the last four weeks. Following an inclusive definition of PA, we applied the overall index. To avoid overreporting and outlier problems, we applied the winsorization technique [50], which cuts down any data points above the 95th percentile. The questionnaire on PAHCO, which has been found reliable and valid in automotive mechatronics and nursing care apprentices [29], was used to assess apprentices’ PAHCO. This questionnaire contains 44 items and eight sub-factors that represent aspects of the three sub-competencies movement competence, control competence, and self-regulation competence.

#### Data analysis

The statistical analyses were performed using IBM SPSS Statistics ver. 25 (IBM, Armonk, USA). A repeated-measures analysis of variance was used to test for between-group differences in changes over time (Group x Time). In addition, main effects between the intervention and control groups (Group) and over time (Time) were analyzed. For differences at baseline, a repeated-measures analysis of covariance was used to adjust for preexisting differences. A significance level of *p* < 0.05 was used for all analyses.

## Results

### Part 1: Sustainability

#### Evaluation of the questionnaire survey

The evaluation of the questionnaire survey on the current implementation status of the multi-component interventions’ different intervention components revealed heterogeneous results in both institutions (see Additional file 1). In the nursing care institution, several intervention components had sustained implementation beyond the course of the project. This held true even though some of them have been temporarily inactive due to COVID-19 protective measures. In the automotive mechatronics institution, different components of the multi-component intervention were initially implemented but were not sustained, regardless of any COVID-19 restrictions. Setting-related differences were also apparent in evaluating the sustainable implementation of intervention components: while the long-term implementation of intervention components was rated as possible in the nursing care institution, this was inconsistent in the automotive mechatronics institution (see Additional file 1). The results of the appraisal of the multi-component interventions’ single components regarding the creation of new capabilities, effectiveness, fit to target group and setting, and value can be found in Additional file 4.

#### Evaluation of the interviews

The evaluation of the interviews via qualitative content analysis revealed 27 different influencing factors on the sustainable implementation of interventions at the outer contextual, inner contextual, intervention, and personal levels of institutions. These four levels represent the coded main categories; the individual influencing factors result from the corresponding subcategories. Table 3 provides an overview of the different levels and the associated influencing factors, including their direction of influence and availability (for more detailed information, see Additional file 5). Key themes from the different levels are further illustrated in the following text.

**Table 3:** Factors influencing sustainability and their availability

Factors influencing sustainability	Automotive mechatronics institution		Nursing care institution	
	**Influence**	**Available**	**Influence**	**Available**
**Outer contextual factors**				
COVID-19 pandemic	-	✓	-	✓
Legal framework				
Law reform of the nursing professions	*n.m.*	*n.m.*	+	✓
Liability	*n.m.*	*n.m.*	-	✓
Openness of the sector to physical activity promotion	*n.m.*	*n.m.*	o	✓
**Inner contextual factors**				
Climate and culture	+	✓	+	✓
Cooperation	o	×	o	✓
Decision-making	o	✓	o	✓
Embedment	+	×	+	✓
Health-promoting leadership	+	×	+	✓
Ownership	*n.m.*	*n.m.*	+	✓
Personnel changes	-	✓	o	✓
Relevance	+	×	+	×
Resources				
Financial	+	×	+	✓
Personnel	+	×	+	✓
Spatial-material	*n.m.*	*n.m.*	+	✓
Temporal	+	✓	+	✓
Strategic planning	+	×	+	✓
**Intervention factors**				
Acceptance	+	✓	+	✓
Effectiveness	+	✓	+	✓
Fit	+	✓	+	✓
Flexibility	*n.m.*	*n.m.*	+	✓
**Personal factors**				
Attitude and mindset	+	×	+	✓
Champion	+	×	+	✓
Commitment	+	×	+	✓
Empowerment	*n.m.*	*n.m.*	+	✓
Qualification	*n.m.*	*n.m.*	+	✓
Support	+	×	+	✓

✓ yes, × no, + positive, *o* neutral, - negative, *n.m.* not mentioned

##### Outer contextual factors

Outer contextual factors relevant to sustained implementation were the COVID-19 pandemic, the legal framework, and the openness of the sector to physical activity promotion. For example, the COVID-19 pandemic negatively affected the implementation of the interventions and the provision of financial resources, as expressed by the head of the school subject of nursing:

“Well, at the moment we’re actually paralyzed by Corona, now as before.”

The legal framework had both a facilitating and a hindering effect on long-term implementation. While the liability issue was associated with difficulties in implementing intervention components, the law reform of the nursing professions [51] had a supportive effect on the implementation of intervention components.

“Actually, this strange generalized nursing apprenticeship, which was so denounced before, was good; that a new curriculum came out, yes. That was actually good for us, because we really decided, as a faculty, that we would write the BuG [lesson] into it. Yes, so it has become a kind of law.” (Head of the nursing education program)

##### Inner contextual factors

Inner contextual factors comprise existing conditions, such as climate and culture, health-promoting leadership, personnel changes, and relevance, as well as created structures or processes, such as cooperation, decision-making, embedment, ownership, resources (i.e., financial, personnel, temporal, spatial-material), and strategic planning. For instance, the lack of relevance resulting from the fact that PA was not the essential topic and mission of the institution was perceived as a barrier.

“The problem is just, [that it is] such an additional topic, a backpack topic, additional tasks.” (Member of the works council)

Also noteworthy, but as a facilitating factor, was the role of health-promoting leadership; its importance was underlined by the head of the nursing education program:

“But of course, the head of the school subject of nursing is always important. The whole thing falls or stands with him. If he says, ‘No, I don’t want that here,’ then it wouldn’t have worked.”

Furthermore, strategic planning, referring to the process of goal setting, monitoring and adjusting, was perceived as a facilitating factor.

“I think you have to set priorities. And I always say that the BuG lesson is the linchpin for me. It has to run. And when it's running quite well, you can think about shifting it, ‘that's running now, now we'll look for a new point where I can start’.” (Head of the nursing education program)

##### Intervention factors

Acceptance, effectiveness, fit, and flexibility emerged as intervention factors promoting sustainability. For example, the head of the nursing education program described the existing acceptance of the interventions as follows:

“I think that it has come across well, certainly to the students. Of course, I find it very interesting that the nurse, as a general type, is not a mover, almost the opposite. And yet it’s actually been well accepted. So, that surprised me even a little bit. But it was really well accepted and the students participated well.”

Additionally, the perceived effectiveness of the intervention is also noteworthy, as highlighted by the coordinator for apprenticeship projects:

“So, I've noticed that many [...] [apprentices] have started to deal with sports activities and join [sports] clubs and become active.”

##### Personal factors

Personal factors associated with the sustainability included attitude and mindset, champion, commitment, empowerment, qualification, and support. For instance, the presence of a champion who took responsibility for the project and managed it with engagement was particularly important.

“[The head of the nursing education program] was there from the very first second. And he was our driving force. Without him, [the project] would not have existed at all.” (Head of the school subject of nursing)

##### Comparison of institutions

Interestingly, there were distinct differences between the number of influencing factors and their availability in automotive mechatronics and nursing care institutions. In the automotive mechatronics institution, the number of influencing factors and the availability of the influencing factors mentioned were lower. For example, factors such as empowerment, flexibility, legal framework, openness of the sector to physical activity promotion, ownership, and qualification were not mentioned in the automotive mechatronics institution, but mentioned in the nursing care institution. Influencing factors that were mentioned but not available in the automotive mechatronics institution include attitude and mindset, champion, commitment, embedment, health-promoting leadership, resources, strategic planning, and support. Although regularity and continuity regarding integration into internal processes and structures were emphasized in both institutions, the embedment was only successful in nursing care.

“Basically, it has to be anchored in the VET process that this is simply one of the obligatory modules among others.” (Member of the works council)

“But the nice thing is that, thanks goodness, we have [the] BuG [lesson] in the curriculum, and that means that it's no longer just a matter of ‘we do [the] BuG [lesson] when we are fun’, but ‘we do [the] BuG [lesson] because it's teaching’. That was so important for us.” (Head of the nursing education program)

Similarly, the factors commitment and support of various actors, such as the target group or workforce, were assessed as facilitating in both the automotive mechatronics and nursing care institutions, but both factors were actually available only in the nursing care institution.

“Yes, [...] and the [rest of the workforce], I think, were quite grateful that the cup had passed from them.” (Member of the works council)

“And I would say that the whole faculty is on board with that, yes.” (Head of the nursing education program)

### Part 2: Effectiveness

The results of the intervention effectiveness presented in Table 4 revealed no significant interaction effects between intervention groups and control groups for changes over time in any of the PA or PAHCO variables observed (*p* > 0.05). Significant time effects were found for increases of the volume of sport activity (*F*(1, 55) = 4.467, *p* = 0.039, η^2^ = 0.075), the volume of overall PA (*F*(1, 55) = 6.382, *p* = 0.014, η^2^ = 0.104), the manageability of balance demands (*F*(1, 53) = 8.483, *p* = 0.005, η^2^ = 0.138), and PA-specific self-efficacy (*F*(1, 54) = 5.524, *p* = 0.022, η^2^ = 0.093) in the automotive mechatronics sample. In the nursing care sample, a significant time effect was detected for a decrease of emotional attitude (*F*(1, 46) = 4.474, *p* = 0.040, η^2^ = 0.089). Although the analyses showed huge variations in the volume of overall PA of automotive mechatronics and nursing care apprentices, ranging from 317.54 ± 279.49 min per day to 554.99 ± 449.94 min per day, altogether these groups possessed a very high amount of PA (see Table 4).

**Table 4:** Changes in PA and PAHCO from pre-intervention (t_0_) to post-intervention (t_1_) by group

Variable	Sample	t_0_	t_1_	Time			Group			Time x Group		
		***M (SD)***	***M (SD)***	***df***	***F***	***p***	***df***	***F***	***p***	***df***	***F***	***p***
**PA**												
Volume of sport activity [minutes/week]	IG-N (*n* = 16)	134.53 (221.43)	123.44 (226.60)	1	0.036	.850	1	0.194	.662	1	0.311	.580
	CG-N (*n* = 32)	152.81 (211.01)	108.13 (159.33)									
	IG-A (*n* = 22)	190.06 (219.59)	209.55 (235.59)	1	4.467	.039*	1	0.021	.885	1	1.769	.189
	CG-A (*n* = 35)	165.40 (193.85)	251.04 (266.59)									
Volume of overall PA [minutes/week]	IG-N (*n* = 16)	392.77 (304.85)	422.19 (466.47)	1	0.313	.579	1	0.308	.582	1	2.881	.097
	CG-N (*n* = 32)	454.76 (338.91)	317.54 (279.49)									
	IG-A (*n* = 22)	422.93 (408.34)	538.24 (418.14)	1	6.382	.014*	1	0.010	.922	1	0.019	.891
	CG-A (*n* = 34)	426.46 (387.54)	554.99 (449.94)									
**PAHCO**												
Manageability of strength demands	IG-N (*n* = 17)	4.49 (0.56)	4.34 (1.00)	1	2.200	.145	1	0.516	.476	1	0.798	.376
	CG-N (*n* = 32)	4.36 (0.68)	4.28 (0.61)									
	IG-A (*n* = 22)	4.44 (0.82)	4.55 (0.53)	1	2.600	.113	1	1.101	.299	1	0.014	.906
	CG-A (*n* = 34)	4.60 (0.57)	4.69 (0.39)									
Manageability of endurance demands	IG-N (*n* = 17)	3.96 (0.83)	3.74 (0.99)	1	2.306	.136	1	1.941	.170	1	3.294	.076
	CG-N (*n* = 32)	4.23 (0.72)	4.33 (0.55)									
	IG-A (*n* = 22)	4.26 (0.78)	4.43 (0.58)	1	2.115	.152	1	1.942	.169	1	0.501	.482
	CG-A (*n* = 34)	4.07 (0.63)	4.13 (0.77)									
Manageability of balance demands	IG-N (*n* = 16)	4.26 (1.02)	4.44 (0.92)	1	0.264	.610	1	0.274	.603	1	0.967	.331
	CG-N (*n* = 32)	4.20 (0.96)	4.63 (0.50)									
	IG-A (*n* = 21)	4.39 (0.62)	4.54 (0.46)	1	8.483	.005*	1	0.075	.786	1	0.568	.454
	CG-A (*n* = 34)	4.37 (0.54)	4.63 (0.42)									
Self-efficacy	IG-N (*n* = 17)	3.61 (1.13)	3.41 (1.19)	1	0.333	.567	1	0.303	.585	1	0.540	.466
	CG-N (*n* = 31)	3.51 (1.04)	3.54 (1.14)									
	IG-A (*n* = 22)	3.88 (0.97)	4.08 (0.84)	1	5.524	.022*	1	0.048	.827	1	0.688	.411
	CG-A (*n* = 34)	3.72 (1.16)	4.13 (1.07)									
Control of physical load	IG-N (*n* = 16)	3.35 (1.13)	3.27 (1.21)	1	0.366	.548	1	0.190	.665	1	1.104	.299
	CG-N (*n* = 32)	3.59 (0.86)	3.73 (0.63)									
	IG-A (*n* = 22)	3.63 (0.78)	3.78 (0.87)	1	0.380	.540	1	0.000	.991	1	1.217	.275
	CG-A (*n* = 35)	3.73 (0.80)	3.69 (0.99)									
Affect regulation	IG-N (*n* = 17)	3.59 (1.38)	3.19 (1.38)	1	0.038	.847	1	0.038	.846	1	0.038	.847
	CG-N (*n* = 32)	3.89 (0.89)	3.60 (1.16)									
	IG-A (*n* = 22)	3.80 (0.97)	3.69 (1.01)	1	0.431	.514	1	0.009	.924	1	1.916	.172
	CG-A (*n* = 34)	3.63 (1.07)	3.91 (1.10)									
Self-control	IG-N (*n* = 17)	3.10 (1.46)	2.55 (1.55)	1	0.116	.735	1	0.111	.740	1	0.186	.668
	CG-N (*n* = 31)	3.32 (1.03)	2.98 (1.20)									
	IG-A (*n* = 22)	3.26 (1.13)	3.32 (1.02)	1	2.469	.122	1	2.819	.099	1	0.945	.335
	CG-A (*n* = 35)	3.58 (0.95)	3.84 (0.94)									
Emotional attitude	IG-N (*n* = 17)	5.00 (1.65)	4.51 (1.99)	1	4.474	.040*	1	1.005	0.321	1	0.964	.331
	CG-N (*n* = 32)	5.50 (1.00)	5.06 (1.47)									
	IG-A (*n* = 22)	5.09 (1.49)	5.02 (1.49)	1	0.300	.586	1	2.036	.159	1	1.070	.305
	CG-A (*n* = 35)	5.41 (1.14)	5.64 (1.24)									

All comparisons in nursing care were adjusted for body mass index

*IG-A* intervention group automotive mechatronics, *IG-N* intervention group nursing care, *CG-A* control group automotive mechatronics, *CG-N* control group nursing care, *PA* physical activity, *PAHCO* physical activity-related health competence

^*^p < .05

## Discussion

The present article provides new and comprehensive insights into the effectiveness and sustainability of participatively developed multi-component interventions in VET, specifically in the automotive mechatronics and nursing care sectors. First, we explored the sustainable implementation of multi-component interventions and the factors that contributed to sustained implementation. Second, two non-randomized controlled trials were used to examine the impact of single components on apprentices’ PA and PAHCO. While we found variability across sites in terms of sustained implementation, no difference was found in effectiveness.

At the institutional level, differences emerged between the implementation statuses of the multi-component interventions. While the long-term implementation of the multi-component intervention could not be registered in the automotive mechatronics institution, single components of the multi-component intervention were still being implemented in the nursing care institution. In this context, many factors influencing the likelihood of sustainable intervention implementation were identified, most of them congruent with the sustainability factors found in recent reviews of the literature [15, 46, 52, 28]: for example, legal framework at the outer contextual level, climate and culture, cooperation, embedment, decision-making, health-promoting leadership, relevance, resources, and strategic planning at the inner contextual level, acceptance, effectiveness, fit, and flexibility at the intervention level, alongside attitude and mindset, champion, qualification, and support at the personal level. In addition, new factors have been identified that have received little attention in the available sustainability literature. For instance, some emerging challenges, such as the outbreak of the COVID-19 pandemic and personnel changes, were mentioned as influencing factors. Further, new influencing factors identified were situational circumstances, such as openness of the sector to PA promotion and engagement, or outcomes, such as the emergence of ownership and empowerment. Although ownership and empowerment have rarely been discussed in the sustainability literature to date [15, 52, 46, 28], both are core concepts in participatory research [24], making their appearance less surprising given our chosen approach.

Other noticeable findings emerged from the differences in the number and availability of influencing factors between automotive mechatronics and nursing care institutions. Thus, not only a higher number of influencing factors were identified in the nursing care institution, but the facilitating factors were also available more frequently. Accordingly, the high number of facilitating factors that were not available could be a possible reason for failed long-term implementation of the intervention in the automotive mechatronics institution. These differences between institutions were most apparent at the inner contextual and personal level, as although approximately equal influencing factors were identified, many of those factors were not available in the automotive mechatronics institution (i.e., attitude and mindset, champion, commitment, embedment, strategic planning, health-promoting leadership, resources, support). Another remarkable result is that ownership at the inner contextual level and empowerment at the personal level were not even mentioned as factors influencing sustainability in automotive mechatronics, although both are core elements of participatory research [24]. As taking responsibility for continuing the intervention is often the consequence of empowerment and ownership [53], the lack of both factors might be a major barrier to successful long-term implementation in the automotive mechatronics institution. However, it remains unclear what contributed to the fact that empowerment and ownership were existent in nursing care, thus increasing the likelihood of sustained intervention implementation, while neither factor was mentioned in automotive mechatronics. With a higher number of influencing factors mentioned, the outer contextual factors seemed to play a greater role in the nursing care institution than in the automotive mechatronics institution. At the intervention level, there were no major differences between the two institutions, which is perhaps unsurprising given the co-creation approach used to develop interventions tailored to the target group and setting.

Examining the various influencing factors explicitly, interrelations between factors became visible, which could be another possible reason for the observed differences in the long-term implementation of the multi-component interventions in both settings. In nursing care institution, for example, the embedment of intervention components was favored by the law reform of the nursing professions. Indeed, this change in the legal framework, coupled with the overall openness of the sector to physical activity promotion, may have created a window of opportunity to place PA promotion in the VET of nursing care [54]. As interventions with relevance to existing aims and policies are easier to implement [55], involving relevant actors from policy and practice in co-creation strategies seems valuable for identifying existing policies, goals, structures, and practices to foster the embedment of an intervention [56, 57]. Furthermore, interrelations between the factors champion and decision-making or health-promoting leadership were found in nursing care; accordingly, the champions were part of the decision-making and health-promoting leadership simultaneously. In the automotive mechatronics institution, in contrast, both the champion and the health-promoting leadership had left the institution due to personnel changes. Since the importance of a champion and leadership in the implementation process predicts implementation success [58–64], losing these important actors due to personnel changes appears to have challenged successful long-term implementation in automotive mechatronics institution. This parallels previous research indicating that personnel changes negatively influenced the long-term implemenation of interventions [64]. Moreover, links between the relevance of the issue of PA promotion and the commitment of actors could be determined in automotive mechatronics; PA promotion was perceived as an additional task and, thus, met with little response and interest from the individuals. These results could also depend on the characteristics of both settings. Therefore, notably, VET in Germany is organized in a dual apprenticeship system combining school-based learning and company-based training. While the development and implementation of the multi-component intervention in the nursing care sector took place at school, in the automotive mechatronics sector, it was conducted at the workplace. In our case, the nursing care school was characterized by a flattened decision-making hierarchy (i.e., the champion was part of the health-promoting leadership and decision-making), and strong existing commitment. In contrast, the automotive company had a more hierarchically decision-making structure, in which the champion was not embedded in the leadership structure, and PA promotion was of low relevance, so that no commitment was demonstrated.

Overall, the differences between the two institutions regarding sustained implementation and the associated influencing factors could also be related to organizational readiness, as the latter is considered a key predictor for successful implementation [65]. According to Scaccia et al. [66], “readiness refers to the extent to which an organization is both willing and able to implement a particular innovation” (p. 485), and it includes the components of the organization’s motivation to adopt an innovation, general organizational capacities, and innovation-specific capacities. Thus, lack of commitment, support, and relevance, alongside the shortage of resources and the absence of a champion could have resulted in lack of readiness to implement the intervention in the automotive mechatronics institution over the short and long-term. In contrast, the actors of the nursing care institution were highly motivated (e.g., commitment, attitude and mindset, support) and utilized existing organizational (e.g., resources) and intervention-specific capacities (e.g., champion), and were thus prepared to implement the interventions in the long-term. In this context, assessing organizational readiness for change from the outset seems worthwhile to identify those institutions that are willing and able to implement the interventions, or otherwise to prepare those institutions that are not yet ready for change by addressing deficits in readiness [67]. Finally, a co-creation approach may be more appropriate for some institutions than others. In the nursing care sector or school setting, participatory intervention development appeared promising, as it was related to sustainable implementation at the institutional level. Even if the readiness for change is present, there must also be a readiness for participation where actors’ participation is important. If an institution is completely closed to the actors’ participation, it would be unsuitable for a co-creation approach [68].

At the individual level, the effectiveness of the multi-component interventions for changing apprentices’ PA behavior and PAHCO, evaluated on the basis of one intervention component per institution, could not be demonstrated. In contrast with previous findings reporting low volumes of PA among nursing care and automotive mechatronics apprentices, our results indicated that these two groups were achieving a very high amount of PA. These results are in line with other recent studies [69–71], each reporting similarly high PA volumes in the automotive mechatronics and nursing care sectors. Regarding the results of the sustainability evaluation, implementation failure could be one of the main reasons for the missing effectiveness in the automotive mechatronics institution. The fact that the interventions implemented were not typical researcher-developed evidence-based interventions implemented in a real-world setting after efficacy had been demonstrated [72–75], but rather co-created interventions based on elements of evidence-based behavior change techniques and tailored to the specifics of the target group and given setting without prior evaluation of their effectiveness under ideal conditions, may be another reason for the lack of intervention effects. This parallels the findings of a recent systematic review reporting that participatively developed interventions tended to improve the relevant psychological factors associated with PA, but not PA levels per se [8]. Although synergizing the scientific world with the real-world is considered a key benefit of co-creation [17], a key challenge appears to be involving the target group to develop target group-specific interventions without neglecting theory and an evidence-based approach.

With respect to the methodological approach, it remains a challenge to evaluate the effectiveness and sustainability of complex multi-component interventions developed in a participatory manner. Although evaluating effectiveness at the individual level and sustainability at the institutional level using a pragmatic evaluation approach provided us with a deep and comprehensive insight into the impact of a co-creation approach, clarifying the role of co-creation requires methodologically complex and elaborate evaluation designs. To meet this claim, a hybrid effectiveness-implementation trial in the context of a cluster-randomized trial seems worthwhile [76, 77]. On the one hand, a hybrid effectiveness-implementation trial allows for a closer look at the interplay between effectiveness and implementation, as information on the intervention effects at the individual level and the effects of the intervention strategy for improving intervention implementation at the institutional level are collected simultaneously, rather than consecutively, with a time lag, as in our case. On the other hand, a cluster-randomized trial provides the opportunity to compare participatively and non-participatively developed interventions and thus clarify the role of co-creation in the effectiveness and implementation or sustainability of these interventions.

### Strengths and limitations

There are several strengths of this study. The comprehensive evaluation using multiple methods allows us to gain new insights into the effectiveness and sustainability of co-created interventions in VET. A scientific evaluation of projects after the end of the project is rare, as funding is often limited to short-term project activities. By comparing co-created multi-component interventions in two contrasting sectors, nursing care and automotive mechatronics, with one delivered in school and one in the workplace, similarities and differences were amplified, allowing for a more complete analysis and interpretation.

However, some limitations also exist. First, we had a moderate response rate to the request for participation in the telephone survey when evaluating sustainability (Part 1); thus, not all perspectives on the current status and appraisal of interventions may be represented. By conducting supplementary interviews and selecting interviewees through a purposeful sampling of information-rich cases, we expected to obtain missing information, but this is impossible to confirm. Second, the interview guide was pilot tested only within the research team, and the transcripts and findings were not provided to interviewees for comments and feedback. Third, we evaluated only one component of the multi-component intervention per setting when evaluating effectiveness (Part 2), limiting our ability to draw conclusions for the entire multi-component intervention. Fourth, we relied exclusively on self-report data for the effectiveness evaluation, which may have resulted in the reporting of higher PA scores due to memory bias. Fifth, the setting-specific conditions meant that we had only a small total sample size and could not conduct a priori power analysis to calculate sample size or randomly assign participants to the intervention or control groups. We were aware of the methodological challenges of evaluating the intervention’s effectiveness in a real-world setting, but we tried to conduct this part of the study in the best possible way by taking a pragmatic evaluation approach.

## Conclusion

Presently, the question of the role of co-creation regarding effective and sustainable PA promotion in VET cannot be answered conclusively. Only in the nursing care sector or school setting, a co-creation approach appeared promising for sustained intervention implementation. However, sustainable intervention implementation also depended on a variety of influencing factors that need to be considered from the outset. Hence, it might be useful to assess setting-specific factors, such as organizational readiness or other important influencing factors, a priori to utilize existing sustainability factors or to focus on deficient factors when developing and implementing interventions and thus to foster their sustainable implementation. To demonstrate effectiveness at the individual level, future intervention projects need to be designed with methodologically elaborate and comprehensive evaluation over a longer time period. In this context, future research should consider cluster-randomized hybrid-implementation trials to clarify both the role and impact of a co-creation approach.

## Supplementary Information

**Additional file 1.** Information on the implementation status of intervention components of the multi-component intervention.

**Additional file 2.** Standards for Reporting Implementation Studies: the StaRI checklist for completion.

**Additional file 3.** COREQ (COnsolidated criteria for REporting Qualitative research) Checklist.

**Additional file 4.** Appraisal of intervention components of the multi-component intervention.

**Additional file 5.** Summary table of factors influencing sustainability.

## Data Availability

The datasets generated for this study are available from the corresponding author upon request.

## Abbreviations

BuG: Ger. “Bewegt und Gesund”, Eng. physical activity and health
COREQ: Consolidated Criteria For Reporting Qualitative Research
CG-A: Control Group Automotive Mechatronics
CG-N: Control Group Nursing Care
IG-A: Intervention Group Automotive Mechatronics
IG-N: Intervention Group Nursing Care
PA: Physical Activity
PAHCO: Physical Activity-Related Health Competence
PArC-AVE: Physical Activity-Related Health Competence In Apprenticeship And Vocational Education
StaRI: Standards For Reporting Implementation Studies
VET: Vocational Education And Training
