# Supplementary material for: Examining the sustainability and effectiveness of co-created physical activity interventions in vocational education and training: a multimethod evaluation
Source: BMC Public Health. 2022 Apr 15;22:765. doi: 10.1186/s12889-022-13133-9 (PMC9011375; doi:10.1186/s12889-022-13133-9)
Supplement: Supplementary file 1 — Additional file 1. Information on the implementation status of intervention components of the multi-component intervention. [file 12889_2022_13133_MOESM1_ESM.pdf]

# Additional file 1: Information on the implementation status of intervention components of the multi-component intervention

**Table 1: Information on the implementation status of the multi-component intervention's components in the nursing care sector (*n* = 4)**

| Intervention component and description | No information (n) | Implementation status | | | | | Sustainability | |
|---|---|---|---|---|---|---|---|---|
| | | Implemented (n) | Planned (n) | Initially implemented, but not perpetuated (n) | Not implemented (n) | Not implemented due to COVID-19 restrictions (n) | Sustainable implementation possible (n) | Sustainable implementation not possible (n) |
| **BuG lesson**<br>Weekly 90-min lesson during regular school hours in each class, covering the theory and practice of PA and health | 0 | 0 | 1 | 0 | 0 | 3 | 4 | 0 |
| **Trainer qualification for teachers**<br>Teachers participate voluntarily in a workshop that qualifies them to conduct the BuG lesson | 0 | 2 | 1 | 0 | 1 | 0 | 4 | 0 |
| **Information for teachers**<br>Information about the project and the developed during a teachers' conference | 0 | 2 | 0 | 0 | 1 | 1 | 3 | 1 |
| **Toolbox**<br>PowerPoint presentation for active breaks with instructions for strength, coordination, and concentration exercises | 0 | 0 | 0 | 2 | 0 | 2 | 4 | 0 |
| **Preserving position of a physical education teacher**<br>Recruitment of a new physical education teacher after the retirement of the current teacher | 1 | 0 | 1 | 0 | 2 | 0 | 2 | 1 |
| **Trainer qualification for students**<br>Students participate voluntarily in a workshop that qualifies them to instruct physical exercises | 3 | 0 | 0 | 0 | 1 | 0 | 1 | 0 |
| **Adaptation of mission statement**<br>PA as part of the mission statement of the VET center | 2 | 2 | 0 | 0 | 0 | 0 | 2 | 0 |

BuG = Ger. "Bewegt und Gesund", Eng. Physical activity and health; PA = physical activity; VET = vocational education and training

**Table 2: Information on the implementation status of the multi-component intervention's components in the automotive mechatronics sector (*n* = 3)**

| Intervention component and description | No information (n) | Implementation status: Implemented (n) | Planned (n) | Initially implemented, but not perpetuated (n) | Not implemented (n) | Not implemented due to COVID-19 restrictions (n) | Sustainability: Sustainable implementation possible (n) | Sustainable implementation not possible (n) |
|---|---|---|---|---|---|---|---|---|
| **Tutoring system**<br>Apprentices participate voluntarily in a workshop enabling them to act as tutors to promote PA and health among their peers | 0 | 0 | 0 | 3 | 0 | 0 | 1 | 2 |
| **Adaptation of "Fit & Healthy Workshop"**<br>Integration of an additional section on PA and health into an existing workshop for first-year apprentices | 0 | 0 | 0 | 3 | 0 | 0 | 1 | 2 |
| **Creation of PA opportunities**<br>Capabilities for PA in the educational and working environment of apprentices, e.g. slots for PA in everyday work routine, exercises at the apprentices' workplace | 1 | 1 | 0 | 1 | 0 | 0 | 2 | 0 |
| **Information for instructors**<br>Information about health-enhancing PA and tutoring workshop during a quarterly instruction | 1 | 0 | 0 | 2 | 0 | 0 | 2 | 0 |
| **Instructor workshop**<br>Automotive mechatronics instructors participate in a workshop on the subject of PA and health | 1 | 0 | 0 | 2 | 0 | 0 | 1 | 1 |
| **Adaptation of works agreement**<br>PA as part of the works agreement of the VET center | 0 | 2 | 0 | 1 | 0 | 0 | 3 | 0 |
| **Adaptation of mission statement**<br>PA as part of the mission statement of the VET center | 2 | 0 | 0 | 0 | 1 | 0 | 0 | 1 |

PA = physical activity; VET = vocational education and training
