# Supplementary material for: Examining the sustainability and effectiveness of co-created physical activity interventions in vocational education and training: a multimethod evaluation
Source: BMC Public Health. 2022 Apr 15;22:765. doi: 10.1186/s12889-022-13133-9 (PMC9011375; doi:10.1186/s12889-022-13133-9)
Supplement: Supplementary file 4 — Additional file 4. Appraisal of intervention components of the multi-component intervention. [file 12889_2022_13133_MOESM4_ESM.pdf]

Additional file 4: Appraisal of intervention components of the multi-component intervention

Table 1: Appraisal of the multi-component intervention's components in the nursing care sector (*n* = 4)

**The intervention component *BuG lesson (Ger. "Bewegt und Gesund", Eng. Physical activity and health)*…**				
… offers the apprentices a new capability to be physically active.	Does not apply	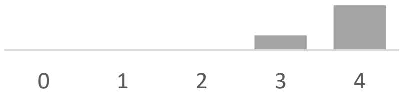	Strongly applies	*n* = 4 *M* = 3.75
… contributes to the apprentices getting more physically active.	Does not apply	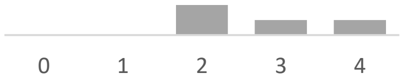	Strongly applies	*n* = 4 *M* = 2,75
… is tailored to the needs and requirements of the apprentices.	Does not apply	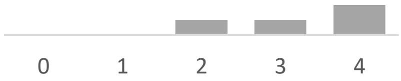	Strongly applies	*n* = 4 *M* = 3.25
… fits to the situation and the organizational conditions in our organization.	Does not apply	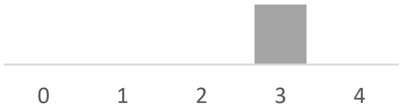	Strongly applies	*n* = 4 *M* = 3.00
… has a great value in our organization compared to other activities/goals.	Does not apply	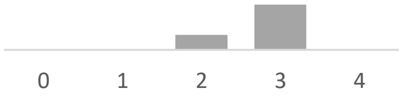	Strongly applies	*n* = 4 *M* = 2.75
**The intervention component *Trainer qualification for teachers*…**				
… offers the apprentices a new capability to be physically active.	Does not apply	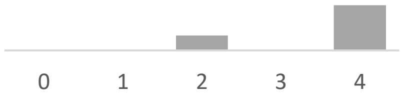	Strongly applies	*n* = 4 *M* = 3.50
… contributes to the apprentices getting more physically active.	Does not apply	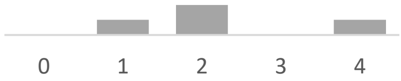	Strongly applies	*n* = 4 *M* = 2.25
… is tailored to the needs and requirements of the apprentices.	Does not apply	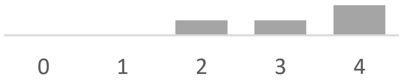	Strongly applies	*n* = 4 *M* = 3.25
… fits to the situation and the organizational conditions in our organization.	Does not apply	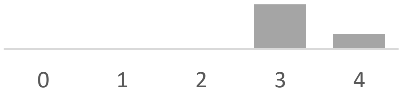	Strongly applies	*n* = 4 *M* = 3.25
… has a great value in our organization compared to other activities/goals.	Does not apply	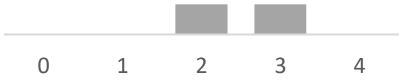	Strongly applies	*n* = 4 *M* = 2.50

The intervention component *Information for teachers*…

… offers the apprentices a new capability to be physically active.

Does not apply

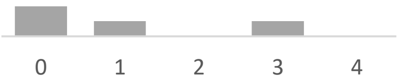

Strongly applies

n = 4
M = 1.00

… contributes to the apprentices getting more physically active.

Does not apply

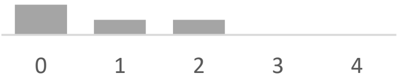

Strongly applies

n = 4
M = 0.75

… is tailored to the needs and requirements of the apprentices.

Does not apply

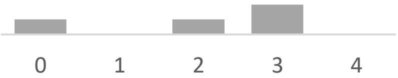

Strongly applies

n = 4
M = 2.00

… fits to the situation and the organizational conditions in our organization.

Does not apply

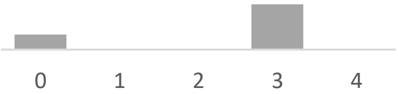

Strongly applies

n = 4
M = 2.25

… has a great value in our organization compared to other activities/goals.

Does not apply

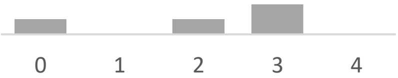

Strongly applies

n = 4
M = 2.00

The intervention component *Toolbox*…

… offers the apprentices a new capability to be physically active.

Does not apply

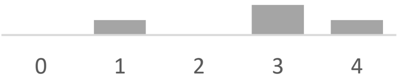

Strongly applies

n = 4
M = 2.75

… contributes to the apprentices getting more physically active.

Does not apply

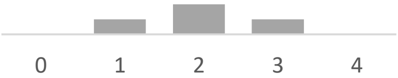

Strongly applies

n = 4
M = 2.00

… is tailored to the needs and requirements of the apprentices.

Does not apply

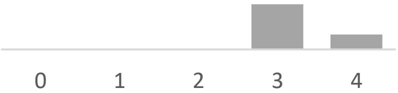

Strongly applies

n = 4
M = 3.25

… fits to the situation and the organizational conditions in our organization.

Does not apply

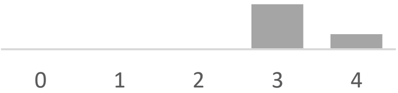

Strongly applies

n = 4
M = 3.25

… has a great value in our organization compared to other activities/goals.

Does not apply

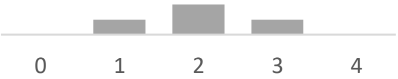

Strongly applies

n = 4
M = 2.00

The intervention component *Preserving position of a physical education teacher…*				
… offers the apprentices a new capability to be physically active.	Does not apply	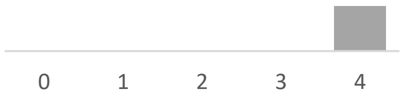	Strongly applies	n = 3 M = 4.00
… contributes to the apprentices getting more physically active.	Does not apply	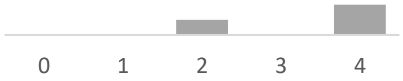	Strongly applies	n = 3 M = 3.33
… is tailored to the needs and requirements of the apprentices.	Does not apply	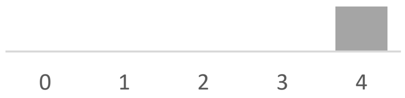	Strongly applies	n = 3 M = 4.00
… fits to the situation and the organizational conditions in our organization.	Does not apply	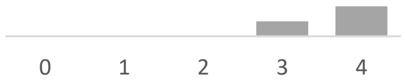	Strongly applies	n = 3 M = 3.67
… has a great value in our organization compared to other activities/goals.	Does not apply	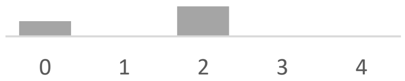	Strongly applies	n = 3 M = 1.33

The intervention component *Trainer qualification for students…*				
… offers the apprentices a new capability to be physically active.	Does not apply	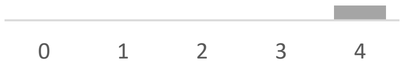	Strongly applies	n = 1 M = 4.00
… contributes to the apprentices getting more physically active.	Does not apply	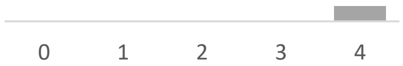	Strongly applies	n = 1 M = 4.00
… is tailored to the needs and requirements of the apprentices.	Does not apply	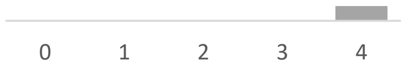	Strongly applies	n = 1 M = 4.00
… fits to the situation and the organizational conditions in our organization.	Does not apply	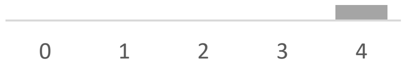	Strongly applies	n = 1 M = 4.00
… has a great value in our organization compared to other activities/goals.	Does not apply	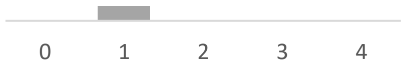	Strongly applies	n = 1 M = 1.00

The intervention component ***Adaption of mission statement*…**				
… offers the apprentices a new capability to be physically active.	Does not apply	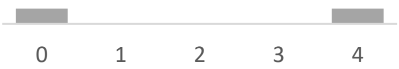	Strongly applies	*n* = 2<br>*M* = 2.00
… contributes to the apprentices getting more physically active.	Does not apply	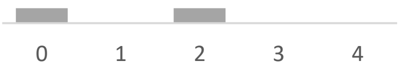	Strongly applies	*n* = 2<br>*M* = 1.00
… is tailored to the needs and requirements of the apprentices.	Does not apply	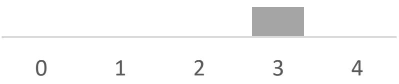	Strongly applies	*n* = 2<br>*M* = 3.00
… fits to the situation and the organizational conditions in our organization.	Does not apply	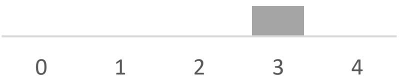	Strongly applies	*n* = 2<br>*M* = 3.00
… has a great value in our organization compared to other activities/goals.	Does not apply	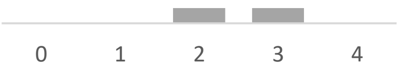	Strongly applies	*n* = 2<br>*M* = 2.50

Table 2: Appraisal of the multi-component intervention's components in the automotive sector (*n* = 3)

**The intervention component *Tutoring system*…**				
… offers the apprentices a new capability to be physically active.	Does not apply	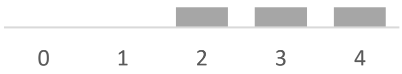	Strongly applies	*n* = 3 *M* = 3.00
… contributes to the apprentices getting more physically active.	Does not apply	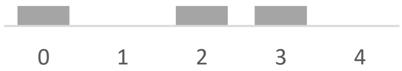	Strongly applies	*n* = 3 *M* = 1.67
… is tailored to the needs and requirements of the apprentices.	Does not apply	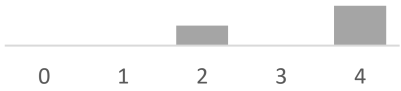	Strongly applies	*n* = 3 *M* = 3.33
… fits to the situation and the organizational conditions in our organization.	Does not apply	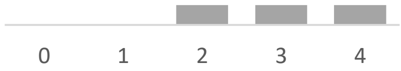	Strongly applies	*n* = 3 *M* = 3.00
… has a great value in our organization compared to other activities/goals.	Does not apply	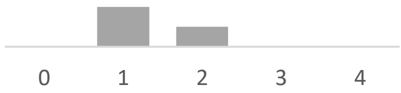	Strongly applies	*n* = 3 *M* = 1.33
**The intervention component *Adoption of "Fit & Healthy Workshop"*…**				
… offers the apprentices a new capability to be physically active.	Does not apply	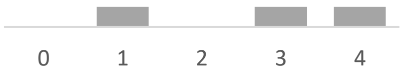	Strongly applies	*n* = 3 *M* = 2.67
… contributes to the apprentices getting more physically active.	Does not apply	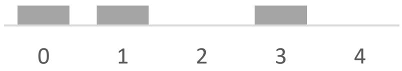	Strongly applies	*n* = 3 *M* = 1.33
… is tailored to the needs and requirements of the apprentices.	Does not apply	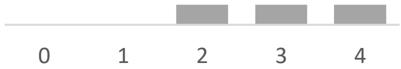	Strongly applies	*n* = 3 *M* = 3.00
… fits to the situation and the organizational conditions in our organization.	Does not apply	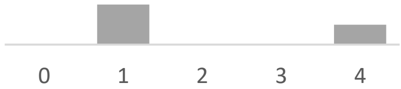	Strongly applies	*n* = 3 *M* = 2.00
… has a great value in our organization compared to other activities/goals.	Does not apply	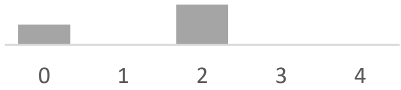	Strongly applies	*n* = 3 *M* = 1.33

The intervention component *Creation of physical activity opportunities*…

… offers the apprentices a new capability to be physically active.

Does not apply

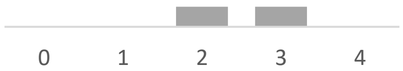

Strongly applies

n = 2
M = 2.50

… contributes to the apprentices getting more physically active.

Does not apply

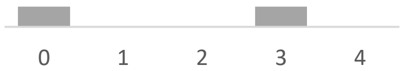

Strongly applies

n = 2
M = 1.50

… is tailored to the needs and requirements of the apprentices.

Does not apply

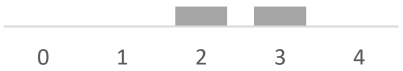

Strongly applies

n = 2
M = 2.50

… fits to the situation and the organizational conditions in our organization.

Does not apply

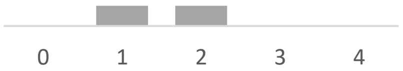

Strongly applies

n = 2
M = 1.50

… has a great value in our organization compared to other activities/goals.

Does not apply

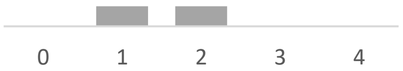

Strongly applies

n = 2
M = 1.50

The intervention component *Information for instructors*…

… offers the apprentices a new capability to be physically active.

Does not apply

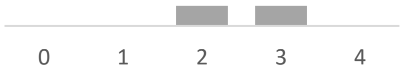

Strongly applies

n = 2
M = 2.50

… contributes to the apprentices getting more physically active.

Does not apply

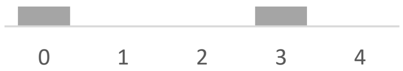

Strongly applies

n = 2
M = 1.50

… is tailored to the needs and requirements of the apprentices.

Does not apply

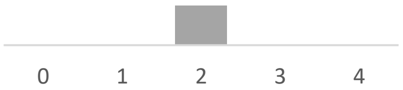

Strongly applies

n = 2
M = 2.00

… fits to the situation and the organizational conditions in our organization.

Does not apply

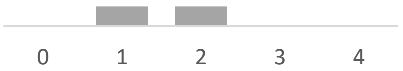

Strongly applies

n = 2
M = 1.50

… has a great value in our organization compared to other activities/goals.

Does not apply

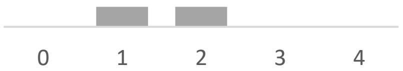

Strongly applies

n = 2
M = 1.50

The intervention component *Instructor workshop*…

… offers the apprentices a new capability to be physically active.

Does not apply

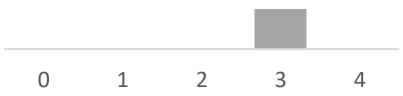

Strongly applies

n = 2
M = 3.00

… contributes to the apprentices getting more physically active.

Does not apply

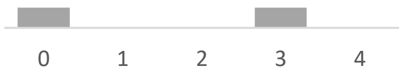

Strongly applies

n = 2
M = 1.50

… is tailored to the needs and requirements of the apprentices.

Does not apply

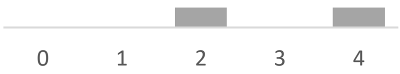

Strongly applies

n = 2
M = 3.00

… fits to the situation and the organizational conditions in our organization.

Does not apply

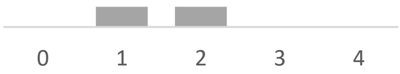

Strongly applies

n = 2
M = 1.50

… has a great value in our organization compared to other activities/goals.

Does not apply

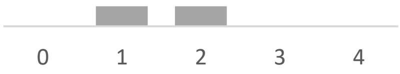

Strongly applies

n = 2
M = 1.50

The intervention component *Adaption of works agreement*…

… offers the apprentices a new capability to be physically active.

Does not apply

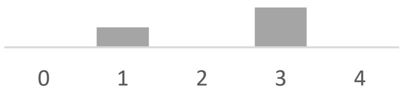

Strongly applies

n = 3
M = 2.33

… contributes to the apprentices getting more physically active.

Does not apply

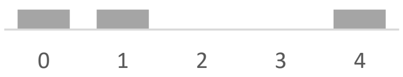

Strongly applies

n = 3
M = 1.67

… is tailored to the needs and requirements of the apprentices.

Does not apply

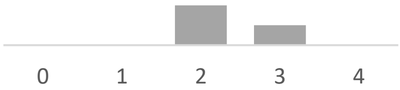

Strongly applies

n = 3
M = 2.33

… fits to the situation and the organizational conditions in our organization.

Does not apply

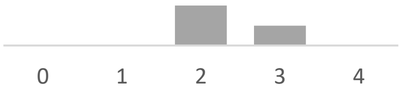

Strongly applies

n = 3
M = 2.33

… has a great value in our organization compared to other activities/goals.

Does not apply

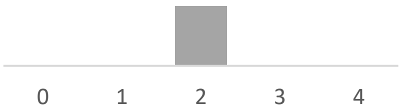

Strongly applies

n = 3
M = 2

**The intervention component *Adaption of mission statement*…**				
… offers the apprentices a new capability to be physically active.	Does not apply	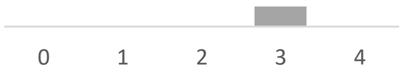	Strongly applies	*n* = 1<br>*M* = 3.00
… contributes to the apprentices getting more physically active.	Does not apply	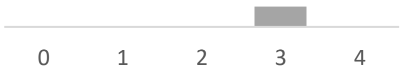	Strongly applies	*n* = 1<br>*M* = 3.00
… is tailored to the needs and requirements of the apprentices.	Does not apply	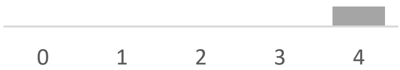	Strongly applies	*n* = 1<br>*M* = 4.00
… fits to the situation and the organizational conditions in our organization.	Does not apply	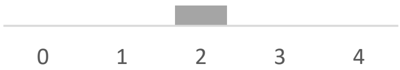	Strongly applies	*n* = 1<br>*M* = 2.00
… has a great value in our organization compared to other activities/goals.	Does not apply	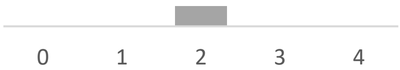	Strongly applies	*n* = 1<br>*M* = 2.00
