# Supplementary material for: Examining the sustainability and effectiveness of co-created physical activity interventions in vocational education and training: a multimethod evaluation
Source: BMC Public Health. 2022 Apr 15;22:765. doi: 10.1186/s12889-022-13133-9 (PMC9011375; doi:10.1186/s12889-022-13133-9)
Supplement: Supplementary file 5 — Additional file 5. Summary table of factors influencing sustainability. [file 12889_2022_13133_MOESM5_ESM.pdf]

Additional file 5: Summary table of factors influencing sustainability

Table 1: Summary table of factors influencing sustainability gathered from interviews with stakeholders from the nursing care (*n* = 2) and automotive mechatronics (*n* = 2) sectors

Factors influencing sustainability	Automotive mechatronic	Nursing care
**Outer contextual factors**		
COVID-19 pandemic	Negative influence on financial resources	Negative influence on implementation status (due to lower relevance, lack of spatial resources) and personal stress
Legal framework	*Not mentioned*	
Law reform of the nursing professions	*Not mentioned*	Positive influence of the change to generalized nursing apprenticeship
Liability	*Not mentioned*	Obstacles due to liability issues
Openness of the sector to physical activity promotion	*Not mentioned*	Existing openness of the nursing care sector to conduct similar projects
**Inner contextual factors**		
Climate and culture	Openness	Openness, no perceived hierarchy, good working atmosphere
Cooperation	External cooperation with service providers and health insurance company was important for intervention implementation and funding, but could not be sustained in the long-term	Internal cooperation with the hospital and other departments is partly problematic due to different interests and needs;<br>External cooperation with service providers was important for intervention implementation;<br>External cooperation with health insurance company for funding was sought, but failed
Decision-making	Head of the automotive education sector made decisions	Heads of the nursing education program and of the school subject of nursing made decisions
Embedment	Embedment has failed;<br>Physical activity must become a compulsory part of the vocational education program	Intervention is part of the curriculum and scheduled with a fixed hourly quota;<br>Regularity and continuity are required
Health-promoting leadership	No one at the management level feels responsible, project was delegated;<br>Involvement of superiors would have been necessary	Headmaster rather uninvolved, but supportive;<br>Heads of the nursing education program and of the school subject of nursing involved and with important function
Ownership	*Not mentioned*	Taking responsibility for the project and its intervention
Personnel changes	Personnel changes among stakeholders and apprentices with a strong negative impact	Personnel changes among stakeholders and apprentices are a challenge, but also an opportunity
Relevance	Physical activity promotion as an additional topic	Physical activity promotion as an additional topic

Resources		
Financial	Funding initially ensured by health insurance, but not in the long-term;<br>Cost-saving initiatives throughout the company	Budget is important;<br>Funding by health insurance company failed, but so far no difficulties with funding (budget was always released)
Personnel	Responsible person is required	Responsible person is available;<br>Coordinator is needed, as various persons are currently taking on this role in addition to their regular tasks
Temporal	Temporal resources at the personnel level were provided	Temporal resources at the personnel level were provided;<br>Temporal resources at the curriculum level are required
Spatial-material	*Not mentioned*	Premises and materials are necessary, both were already available;<br>Premises not always freely accessible;<br>Some new material acquisitions
Strategic planning	Aligning and controlling did not take place	Focusing and prioritizing intervention, setting clear goals, constantly aligning and refocusing
**Intervention factors**		
Acceptance	Intervention was considered good	Not all intervention components are equally accepted by apprentices (preferences exist), but overall well accepted
Effectiveness	Intervention's effectiveness was rated positively	Intervention's effectiveness was rated positively
Fit	Intervention assessed as appropriate for the target group	Intervention assessed as appropriate for both the target group and setting
Flexibility	*Not mentioned*	Flexibility in implementing the BuG lesson (Ger. "Bewegt und Gesund", Eng. Physical activity and health)
**Personal factors**		
Attitude and mindset	Trainers who exemplify it should endorse it and have an affinity for sports	Teacher have an affinity for sports, are uncomplicated, pragmatic and self-confident (hands-on attitude);<br>Personal preferences for individual intervention components exist;<br>Fearfulness due to the liability issue was existent, but could be removed through qualification and information intervention components
Champion	Occupational physician was a champion	Heads of the nursing education program and of the school subject of nursing are champions
Commitment	No commitment, people seem annoyed;<br>Acceptance and awareness of the importance of physical activity needs to be created	High level of commitment (on the part of the teachers and apprentices)

Empowerment	*Not mentioned*	Confidence to continue the project and its intervention even without external support by researchers
Qualification	*Not mentioned*	Physical activity promotion is not an unknown topic;<br>Necessary qualification (i.e., sports scientist) and own experience in promoting physical activity at school were available and helpful
Support	Support of youth apprentices' representatives;<br>No support of trainers	Active support of fellow teachers

This table was created based on the interview results. The individual factors influencing sustainability represent the subcategories and the respective levels the main categories of the structuring content analysis.
